# A Cautionary Tale on Atrial Capture Management, Biventricular Pacing, and Recurrent Asystole

**DOI:** 10.19102/icrm.2019.101001

**Published:** 2019-10-15

**Authors:** Christopher L. Johnsrude, Kelvin C. Lau

**Affiliations:** ^1^Division of Pediatric Cardiology, Department of Pediatrics, University of Louisville, Louisville, KY, USA

**Keywords:** Asystole, biventricular pacemaker, capture management, congenital heart disease, epicardial leads

## Abstract

Capture management algorithms in current cardiac implantable electronic devices (CIEDs) can enhance device performance and battery longevity. Although generally safe, these algorithms have on rare occasions been implicated in the onset of significant complications, especially in pacemaker-dependent patients. CIEDs implanted in patients with postoperative congenital heart disease (CHD) often require epicardial pacing leads rather than transvenous leads; unfortunately, epicardial leads can experience higher rates of malfunction. We herein report on a young adult with a status of postoperative CHD and complete atrioventricular block following implantation of a epicardial dual-chamber cardiac resynchronization therapy pacemaker (CRT-P; Consulta^®^; Medtronic, Minneapolis, MN, USA) who developed frequent periods of asystole after malfunction of one of the ventricular leads. The underlying cause of asystole was found to be due to the atrial capture management (ACM) algorithm of the CRT-P device, temporarily converting biventricular to right ventricular–only pacing as part of the algorithm. This case highlights implications of the ACM algorithm in devices with a similar platform for pacemaker-dependent patients.

## Introduction

Capture management algorithms have been developed for permanent pacemakers, cardiac resynchronization therapy (CRT) devices, and cardioverter-defibrillators to enhance battery longevity and device performance.^1–6^ Although generally safe, significant complications have been reported in patients who are pacemaker-dependent.^7,8^ Patients with postoperative congenital heart disease (CHD) often require epicardial rather than transvenous pacing leads,^9^ and CRT systems are now being implanted more frequently in these individuals.^10–12^ Unfortunately, epicardial leads can have higher rates of malfunction as compared with transvenous leads.^13,14^ We herein report the case of a young adult with postoperative CHD and complete atrioventricular (AV) block (CAVB) following the implantation of an epicardial dual-chamber CRT pacemaker who developed frequent periods of asystole due to the atrial capture management (ACM) algorithm of his CRT pacemaker and concomitant malfunction of one of the epicardial ventricular leads.

## Case report

Our patient was a 25-year-old male with tricuspid atresia and normally related great arteries (type Ic) who had undergone pulmonary artery banding at one month of age and bidirectional Glenn anastomosis at 16 months of age. Extracardiac Fontan operation and subaortic resection were performed at 5.5 years of age, complicated by postsurgical CAVB and left middle cerebral artery stroke. An epicardial dual-chamber pacemaker was implanted at that time. Except for requiring chronic treatment of a seizure disorder, the patient had been otherwise clinically stable from this point onward. In subsequent years, however, his atrial and ventricular epicardial leads failed, prompting multiple surgical revisions via sternotomy and thoracotomy. Serial outpatient evaluations revealed sinus node dysfunction, persistent CAVB, and progressive pacemaker-dependence with only intermittent intrinsic escape rhythm. His last pacemaker surgery at three years prior to this most recent admission included an upgrade to an epicardial dual-chamber CRT pacing system to provide an extra ventricular lead given his prior lead failures and pacemaker-dependence^15^ and to potentially preserve ventricular systolic performance, as he was 100% ventricularly paced. The older model 4965 (Capsure^®^ Epi; Medtronic, Minneapolis, MN, USA) right ventricular (RV) lead was incorporated in this system, as its performance at surgery was judged to be acceptable (capture threshold: 1.5 V at 0.4 ms, impedance: 354 Ω). Therefore, his pacing system at the time of this admission consisted of a Consulta^®^ CRT pacemaker (CRT-P) generator (Medtronic, Minneapolis, MN, USA), the 13-year-old unipolar lead connected to the RV port, and three-year-old bipolar epicardial leads (model 4968 Capsure^®^ Epi; Medtronic, Minneapolis, MN, USA) on the left atrial appendage (LAA) and left ventricle (LV). About two years prior to the current admission, however, the older RV lead had developed markedly elevated capture threshold and lead impedance. As this device platform does not permit reprogramming to entirely isolate this lead, the RV channel was programmed to minimal output (0.5 V at 0.03 ms) to minimize battery drain and maximal sensing threshold (11.3 mV) to reduce the chance for oversensing of nonphysiologic electrical transients. Fortunately, echocardiography performed with the device essentially programmed with LV pacing only indicated the existence of preserved ventricular systolic function.

At 25 years of age, the patient was referred by his primary cardiologist for cardiac catheterization to evaluate Fontan anatomy and physiology, to be followed by hospitalization to evaluate increasingly frequent neurological “spells” possibly related to his remote perioperative stroke. Catheterization showed acceptable Fontan pressures and mild left pulmonary artery stenosis that was treated uneventfully by balloon angioplasty and stent placement. Following catheterization, he was admitted to a telemetry unit in an adjacent adult community hospital for neurology consultation.

**Figure 1:**
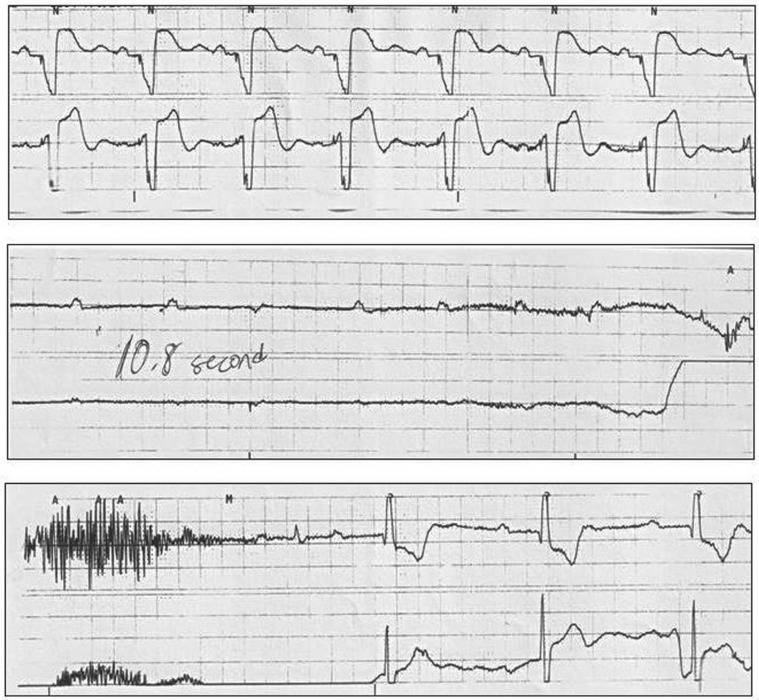
Inpatient telemetry recording of a 25-year-old male patient with complex postoperative CHD and CAVB following dual-chamber biventricular pacemaker showing appropriate DDD pacemaker function followed abruptly by 11 seconds of ventricular asystole and then slow ventricular escape beats. During asystole, note the slightly faster atrial rates with mildly variable P-wave morphologies and no clear atrial pacing spikes (see text for explanation).

On the night of catheterization, continuous electrocardiogram telemetry recorded several periods of asystole lasting between 10 seconds and 12 seconds in the early morning hours **(Figure 1)**. Device interrogation confirmed that the device was programmed in the DDD mode (lower rate: 60 ppm; upper rate: 150 ppm) with 87% atrial sensing–ventricular pacing and 13% AV pacing. Bedside interrogation revealed stable LAA and LV lead characteristics (LAA capture threshold: 1 V at 0.4 ms, sensing: 1.3 mV, and impedance: 532 Ω; LV capture threshold: 0.875 V at 0.4 ms and impedance: 760 Ω). The RV lead characteristics were as expected, with noncapture at all outputs, 5.3 mV sensing of very rare intrinsic beats, and an impedance of 1,957 Ω. Further assessment showed no baseline “noise” or evidence of electrical transients on the RV channel nor monitored ventricular events (with VT zone programmed to > 154 bpm). Capture management algorithms were programmed as follows: atrial to “adaptive,” RV to “off,” and LV to “monitor.” Automated device diagnostics indicated a “possible high LV threshold” recorded the day before this interrogation and an inability “to measure LV thresholds in the last seven days.”

Given these findings and this patient’s clinical history, we suspected his episodic asystole might reflect LV lead failure; other considerations included intermittent RV oversensing and automated device algorithms. He was transferred to an intensive care unit and prepared for emergency implantation of a new ventricular lead, a proposition that would require extensive open-chest surgery. The output on the LV channel was increased and intravenous dopamine was made available for cardioaccelerator therapy if necessary. Overnight, similar pauses were again seen on telemetry. The device mode was then reprogrammed to VOO, and subsequent inpatient telemetry showed no more pauses.

Close review of telemetry recordings during asystolic periods **(Figure 1)** confirmed pauses occurring in the early morning hours during a period of slightly faster atrial rhythms with mildly varying P-wave morphologies; atrial pacing spikes were not clearly consistent at this time. ACM algorithms of the Medtronic Consulta^®^ CRT-P device (Medtronic, Minneapolis, MN, USA) were nominally performed around 1:00 AM, beginning with atrial pacing slightly faster than the prevailing sinus rhythm to evaluate responses to varying outputs. In patients with AVB, atrial capture is confirmed by atrial chamber reset (ACR). After review of the manufacturer’s manual, we learned that, during ACR, the device’s baseline biventricular pacing temporarily converts to RV-only pacing to enhance the detection of atrial signals. As our patient’s RV lead had ventricular noncapture, we surmised the ACR algorithms could be responsible for our patient’s intermittent asystole.

The device was programmed back to DDD and ACM was turned off. Inpatient telemetry over the next five days showed appropriate pacemaker performance and no pauses. The LV channel was programmed to a unipolar configuration given a lower capture threshold as compared with a bipolar configuration, and recent automated LV capture management had indicated a possibly elevated LV capture threshold (although this was not confirmed by bedside testing).

Of note, formal neurology consultation concluded that the patient’s recent “spells” were not consistent with intermittent asystole but instead reflected progression in chronic encephalopathy and seizure disorder. Our patient responded to medical treatment.

## Discussion

This case illustrates a previously unreported example whereby an ACM algorithm in a biventricular pacemaker introduced significant consequences in a pacemaker-dependent patient with CAVB and RV lead failure. In our patient, upgrading to a dual-chamber biventricular system was intended to provide a safety net given his pacemaker-dependence and history of multiple epicardial lead failures as well as the possible benefits of cardiac resynchronization; this strategy, however, turned out to be a double-edged sword. Although he benefited from having a second ventricular lead, which ultimately obviated the need for surgical revision, leaving the ACM algorithm engaged caused frequent periods of asystole in the early morning hours after his RV lead failed to capture. Indeed, he may have been experiencing frequent periods of asystole for many months, but this was only discovered incidentally by inpatient telemetry during evaluation for another reason. This important ACM-related problem had gone unnoticed for several reasons, including that periods of asystole occurred during typical sleeping hours, serial device interrogations alone would not reveal the problem, and outpatient ambulatory electrocardiogram recordings are not routinely obtained in similar patients unless specific questions arise from patient symptoms or worrisome findings on device interrogation.

Over recent decades, device manufacturers have developed several approaches to minimize battery drain by facilitating lower paced outputs in a reliable and safe manner.^1,2,4–6^ Automated capture management algorithms require precise engineering to confirm pacing-induced myocardial depolarization, either by discerning evoked responses from polarization afterpotentials in ventricular leads or by evaluating AV conduction or resetting of intrinsic sinus rhythm in atrial leads. These algorithms have been shown to be generally accurate and safe in diverse adult and pediatric patient populations across a broad spectrum of pacing lead types and implant locations. However, a few case reports reveal such algorithms can place patients with AVB at risk for serious consequences. Ho et al.^8^ described a patient with AVB status after implantation of a Concerto^®^ CRT defibrillator (Medtronic, Minneapolis, MN, USA) for ventricular fibrillation who developed intermittent ventricular nonoutput due to cross-talk inhibition from atrial afterdepolarizations that were oversensed on the ventricular channel during automated LV capture management algorithms. Separately, Suri et al.^7^ reported on a pacemaker-dependent patient with a St. Jude Pacesetter Affinity DR generator (Abbott Laboratories, Chicago, IL, USA) who developed 10-second periods of ventricular asystole during ventricular autocapture related to increasing lead capture threshold and an internal “housekeeping function” in the device designed to improve the estimation of battery longevity.

Our case revealed recurrent periods of ventricular asystole caused by an underappreciated feature of the Consulta^®^ CRT-P device’s ACM algorithm in patients with AV block; notably, this algorithm is found in multiple Medtronic devices with a similar platform. ACM algorithms in the Consulta^®^ CRT-P device (Medtronic, Minneapolis, MN, USA) are performed only when the device is programmed DDD or DDDR. Because prior studies have shown diurnal variations in capture thresholds with the highest thresholds in the early morning hours,^16^ device diagnostics including ACM are conventionally performed around 1:00 AM. Once the device determines the patient’s rhythm is stable, ACM threshold testing is initiated by pacing the atrium slightly faster than the prevailing sinus rate. This device uses two distinct methods to determine atrial capture thresholds. In patients with stable 1:1 AV conduction during atrial pacing, the AV Conduction algorithm is used, which monitors responses recorded on the ventricular channel to determine the loss of atrial capture. In patients with AV block, the ACR method is employed. ACR relies on expected electrophysiological resetting of the sinus node to determine a loss of atrial capture; when an atrial sensed event occurs soon after an impulse is delivered (within the atrial refractory period), the algorithm “decides” that the sinus node had not been reset by that impulse and therefore atrial myocardium has not been captured. If ACM is programmed to “adaptive,” the device automatically adjusts pacing outputs according to responses to changed outputs, while, when programmed to “monitor,” similar automated maneuvers are performed and responses tracked, but no reprogramming occurs. Importantly, during ACM threshold testing, the Consulta^®^ CRT-P device (Medtronic, Minneapolis, MN, USA) temporarily disables biventricular pacing, converting to RV-pacing only; this circumstance caused ventricular noncapture and asystole in our patient due to pre-existing RV lead failure. Turning ACM off eliminated this temporary RV-only pacing and associated asystole in our patient.

Despite several large case series demonstrating accurate and reliable performance of this algorithm in similar Medtronic devices,^3,4^ this problem has not been previously reported. The lack of detection of this problem in these case series may reflect limitations similar to those described above for our patient but may also reflect the superior long-term performance of transvenous leads in older sedentary adults versus epicardial leads in younger and more active patients. However, after our recent institutional understanding of this issue, an older adult with a transvenous biventricular ICD and similar platform was found to also have this problem during incidental ambulatory monitoring, and this was promptly corrected by turning off ACM.

In summary, our case highlights that the ACM algorithm in the Consulta^®^ CRT-P device (Medtronic, Minneapolis, MN, USA) can cause periods of ventricular asystole in patients with AVB and RV lead failure due to temporary conversion from biventricular to RV-only pacing as part of the ACR subalgorithm. This may be occurring in other patients with similar circumstances or in those with RV pacing output essentially disabled for other reasons, whether or not they are postoperative CHD with epicardial leads. We recommend that, for new implants of this device in similar patients, clinicians consider inserting the newest (or most reliable) ventricular lead into the RV port. For devices currently in use, clinicians should not be reassured by the use of redundant ventricular leads but instead should closely monitor RV lead performance and examine the risk–benefit profile of ACM for each patient. Periodic ambulatory electrocardiogram recordings may provide helpful surveillance. As for managing devices from other manufacturers, clinicians should consult user manuals, field representatives, or company engineers to determine the possibility that this problem might be occurring in some of their patients.
